# Detection, differentiation and localization of replay attack and false data injection attack based on random matrix

**DOI:** 10.1038/s41598-024-52954-z

**Published:** 2024-02-02

**Authors:** Yuehao Shen, Zhijun Qin

**Affiliations:** 1 Guangxi Key Laboratory of Power System Optimization and Energy Technology, Guangxi University, No. 100, Daxue Road, Xixiangtang District, Nanning, 540003 Guangxi People’s Republic of China; 2 School of Electrical Engineering, Guangxi University, No. 100, Daxue Road, Xixiangtang District, Nanning, 540003 Guangxi People’s Republic of China

**Keywords:** Electrical and electronic engineering, Information technology, Computer science

## Abstract

Replay attack and false data injection attack (FDIA) are two common types of cyber-attacks against supervisory control and data acquisition systems, aiming to disrupt the normal operation of the power system by falsifying meter measurements. In this paper, we proposed a systematic methodology to defend hybrid attack with both replay attack and FDIA. Specifically, we propose a detection method applying random matrix theory to: (1) detect the hybrid attack on static state estimation, and (2) distinguish FDIA from replay attack as well as localize falsified measurements. Firstly, short-term forecast on load and renewable power generation is conducted to obtain the predicted measurements. Secondly, random variables are calculated by differentiating the forecasting measurements and real-time measurements. A random matrix is consequently constructed with the above random variables. Thirdly, hybrid attacks are detected by the changes of the linear eigenvalue statistics of the random matrix obtained by the sliding time window. More importantly, a novel multi-label classifier to distinguish replay attack from FDIA is designed to localize FDIA by combining SVD decomposition and eigenvalue analysis with convolutional neural network (SVD-CNN). Finally, comprehensive simulations on the IEEE 14-bus system and IEEE 57-bus system are provided to validate the performance of the proposed method. It is shown that the proposed detection method has strong detection ability by filtering measurement noise. Moreover, the proposed SVD-CNN improves the accuracy in FDIA localization.

## Introduction

With the development of power systems toward the cyber-physical power system (CPPS) with highly integrated communication sub-systems, the vulnerability of power systems in the face of cyber-attacks has greatly increased. In CPPS, supervisory control and data acquisition (SCADA) system is responsible for monitoring and controlling numerous sensors and actuators and is susceptible to cyber-attacks. Attackers may invade the remote terminal units (RTUs) through physical access, network intrusion, malware, deception, or disguise in order to launch cyber attacks. Replay attacks and false data injection attacks (FDIA) are two common types of cyber-attacks against SCADA systems, which aim to disrupt the normal operation of the system by modifying meter measurements. Specifically, the former will intrude RTUS and override real-time measurements with historical data tagged by new timestamps, while the latter modifies real-time measurements by injecting carefully constructed biases.

Mo and Sinopoli^1,2^ first introduced the replay attack on the cyber-physical system and analyzed the effect of replay attacks on a control system. To launch the replay attack, the attackers will falsify all the real-time measurements by the recorded historical measurements. For the replay attack against CPPS, the work in^3^ proposed an efficient detection scheme based on a periodic injection of artificial noise to the system. Ref.^4^ proposed a detection method based on a game-theoretic of designing noise into the control input, considering both control performance and detection precision. Based on^1^, Zhao, Wang and Li proposed a novel control strategy to balance control efficiency and detection precision^5^. As shown above, most detection methods for replay attacks are developed from the perspective of dynamic state estimation based on Kalman filters.

On the other hand, FDIA has gained much research attention since proposed by Liu^6^ in 2009. By injecting carefully constructed bias into meter measurements, FDIA can avoid being detected by bad data detection (BDD) or other detection algorithms in various application contexts^7,8^. FDIA can cause large deviation in state estimation and create adverse impact on economics^9,10^ and stability^11,12^ of power systems. Recently, various detection methods for FDIA have been proposed, which can be primarily categorized into two groups. One is classification algorithms based on machine learning, and the other is based on time series analysis. The former trains a classifier with normal data and falsified data by cyber-attacks, and then identifies attacks using the obtained classifier. For example, minimization of the maximum distance between two hyperplanes^13^, multi-label convolutional neural network (CNN)^14^, Graph Auto-Encoder (GAE) with Residual Neural Network (ResNet)^15^ have been comprehensively studied to detect and localize measurements falsified by FDIA. For the latter type of methods, Ref.^16,17^ achieve the detection of FDIA by examining the difference between probability distributions obtained from measurement variations and have achieved high detection probability. By contrast, some other detection methods are proposed to analyze the consistency between real-time measurements and predicted measurements, and use outliers to identify falsified data. Kalman filter is a widely used method to obtain the predicted dynamic states, and usually used to detect FDIA targeting on power system dynamics^18,19^. For FDIA on static state estimation, the predicted measurements were obtained with load forecasting and steady-state power flow calculation^20^. Subsequently, the predicted measurements were compared with real-time measurements to detect FDIA.

Existing methods mainly focus on detecting one certain type without the ability to detect hybrid attack with both types. Since it is impractical to assume the types of cyber-attack are known in advance from the defender’s perspective, it is the key step to detect and differentiate replay attacks and FDIAs, to implement subsequent localization and measurement recovery. The fundamental challenges in detection of replay attack are twofold, besides that it can bypass BDD. First, Kalman filter methods are not applicable for replay attack targeting on static state estimation, since the quasi-steady condition does not hold. Second, the falsified measurements are directly taken from the historical dataset, having the same numerical characteristics as normal data. Therefore, the classifier-based anomaly detection algorithms may not work well. To address the above challenges, inspired by^20^, we aim to obtain the predicted measurements as ground truth to detect both replay attack and FDIA on static state estimation after bypassing the possible changes in the operating condition of the power system, such as short-circuit faults by the method in^21^. Different from^20^, we place our focus to address the key challenges in the context of high noise of measurements.

It is noticed that random matrix theory (RMT) provides a good method to analyze large datasets with independent and identically distributed (i.i.d.) noise. High-dimensional analysis and construction of high-dimensional statistics based on RMT can quantify data correlation, which is equivalent to a noise filter in some sense. Therefore, RMT has been widely used for voltage stability analysis and anomaly detection in power systems^22–24^. Morever, Ref.^8^ proposed a method based on RMT to construct FDIA attack strategy with measurement data traces collected over a limited period of time.

Inspired by the merits of RMT, we aim to develop a method to detect replay attack and FDIA considering the noise in real-time measurements. Further to distinguish between the two types of attacks and localize FDIA, inspired by the sensitivity of matrix eigenvectors to specific eigenvalues^24^ and multi-label classification^14^, we combined matrix SVD decomposition with multi-label CNN (SVD-CNN) to enhance the accuracy of differentiation and localization.

The major contributions of this paper are twofold. (1) We propose a two-stage method to detect replay attack and FDIA based on RMT. In the first stage, the short-term prediction on load and renewable power generation is conducted. With the prediction, power flow calculation is employed to obtain the short-term prediction of measurements. In the second stage, RMT is used to construct higher-order statistics, namely LES, to analyze the difference between real-time measurements and predicted measurements, as well as the chronological correlation of the difference. To our knowledge, the proposed method is the first attempt using RMT to detect replay attack and FDIA. (2) A novel CNN classifier is developed with two purposes, namely, to distinguish replay attack from FDIA, and to localize the falsified measurement by FDIA. Particularly, the salient feature of the proposed CNN (SVD-CNN) is that the feature extraction layer of CNN is a well-designed sensitive location indicator based on matrix SVD decomposition and eigenvalue analysis.

The remainder of this paper is organized as follows. We briefly introduce FDIA and replay attack model in “Section FDIA and replay attack model”. In “Section Basics of random matrix theory and linear eigenvalue statistics”, we introduce the basic concepts of RMT. Our proposed detection, differentiation and localization methodology is introduced in “Section Detection, differentiation and localization of hybrid attack”. The case studies are presented in “Section Case studies”. Finally, this paper is concluded in “Section Conclusion”.

## FDIA and replay attack model

The measurement model of the power system used in this paper is based on the DC power flow equation given by

1 $$\begin{aligned} {\varvec{z}} = {\varvec{Hx}} + {\varvec{e}}, \end{aligned}$$

where ${{\varvec{x}}} = {[{x_1},{x_2},\ldots ,{x_n}]^T} \in {\mathbb {R}}^n$ represents the state variables, i.e., the bus phase angles; ${{\varvec{z}}} = {[{z_1},{z_2},\ldots {z_m}]^T} \in {\mathbb {R}}^m$ are the measurements, namely, the active power injection of buses and the branch flows; ${{\varvec{H}}} = ({h_{i,j}}) \in {\mathbb {R}}^{m \times n}$ is the Jacobian matrix of power flow equations; ${{\varvec{e}}} = {[{e_1},{e_2},\ldots {e_m}]^T}\in {\mathbb {R}}^m$ represents measurement errors.

### FDIA model

Based on^6^, the falsified measurement can be represented by

2 $$\begin{aligned} {{{\varvec{z}}}_a} = {{\varvec{z}}} + {{\varvec{a}}} = {{\varvec{z}}} + {{\varvec{Hc}}}, \end{aligned}$$

where ${{\varvec{a}}}$ is the inserted bias; ${{\varvec{c}}}$ represent the expected state change caused by the inserted measurement bias. In other words, the following equation holds,

3 $$\begin{aligned} {{{\varvec{x}}}_a} = {{\varvec{x}}} + {{\varvec{c}}}. \end{aligned}$$

Once the condition ${{\varvec{a}}} = {{\varvec{Hc}}}$ holds, the pair $({{{\varvec{x}}}_a},{{{\varvec{z}}}_a})$ will pass BDD. It is noted that to successfully construct FDIA, the attackers need to know at least part of the topology information (namely the element of ${{\varvec{H}}}$ ) of the power grid^25^.

### Replay attack model

Different from FDIA, the replay attack is not dependent on the information of ${{\varvec{H}}}$. By contrast, it is assumed that the attack can access the historical measurements and can falsify all the real-time measurements with historical measurements.

Suppose the replay attack is launched at time interval *t* . The real-time measurements are falsified by

4 $$\begin{aligned} {{{\varvec{z}}}_{a,t}} = {{{\varvec{z}}}_{t - \Delta t}}, \end{aligned}$$

where ${{{\varvec{z}}}_{a,t}}$ represents the falsified real-time measurement at time interval *t* ; ${{{\varvec{z}}}_{t - \Delta t}}$ is the historical measurement at time interval $t - \Delta t$ . Obviously, after static state estimation, the real-time state will equal to the state at time interval $t - \Delta t$ . Therefore, the equivalent inserted measurement bias is given by

5 $$\begin{aligned} {{{\varvec{a}}}_t} = {{{\varvec{z}}}_t} - {{{\varvec{z}}}_{a,t}} = {{{\varvec{z}}}_t} - {{{\varvec{z}}}_{t - \Delta t}}. \end{aligned}$$

In the above equation, ${{{\varvec{z}}}_t}$ is the correct measurement at time interval *t* . Accordingly, the falsified state variable is given by

6 $$\begin{aligned} {{{\varvec{x}}}_{a,t}} = {{{\varvec{x}}}_{t - \Delta t}}, \end{aligned}$$

where ${{{\varvec{x}}}_{a,t}}$ represents the falsified real-time state variable; ${{{\varvec{x}}}_{t - \Delta t}}$ is the state variable at time interval $t - \Delta t$. Apparently, if $({{{\varvec{x}}}_{t - \Delta t}},{{{\varvec{z}}}_{t - \Delta t}})$ can pass BDD, $({{{\varvec{x}}}_{a,t}},{{{\varvec{z}}}_{a,t}})$ after the replay attack can also pass BDD.

## Basics of random matrix theory and linear eigenvalue statistics

Our proposed detection method for hybrid attacks is based on RMT. Therefore, for sake of self-containedness, the fundamental concepts of RMT and linear eigenvalue statistics are briefly introduced as follows.

### Marchenko-Pastur law

Marchenko-Pastur Law (M-P Law) is a basic theorem in RMT, describing the asymptotic behavior of singular values of large rectangular random matrices. Let us consider a Hermitian matrix ${{\varvec{M}}}$ whose entries are i.i.d. with zero mean and bounded variance ${\sigma ^2}$. When the number of rows *N* and the number of columns *T* tend to infinity with a fixed ratio $s = N/T \in (0,1]$, the empirical spectrum density (ESD) of the corresponding sample covariance matrix $\sum$ converges to the distribution represented as the following function

7 $$\begin{aligned} f(\lambda ) = \left\{ \begin{array}{l} \frac{1}{{2\pi \lambda s{\sigma ^2}}}\sqrt{(b - \lambda )(\lambda - a)},\;a \le \lambda \le b\\ 0,\;{\textrm{otherwise}} \end{array} \right. , \end{aligned}$$

where $$a = {\sigma ^2}{(1 - \sqrt{s} )^2}$$, $$b = {\sigma ^2}{(1 + \sqrt{s} )^2}$$.

**Figure 1:**
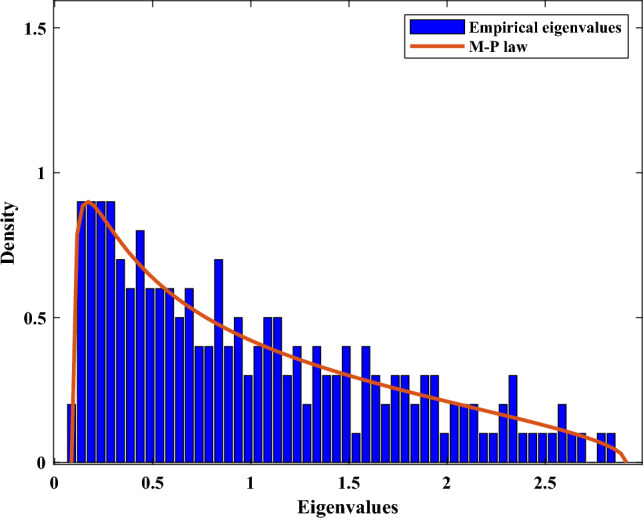
ESD and M-P law.

For a matrix of a finite size (e.g., with tens to hundreds of rows and columns), Eq. (7) is also hold with high accuracy^23^. As shown in Fig. 1, the ESD converges to the theoretical curve of M-P law when $N = 200$ and $T = 400$ .

### Linear eigenvalue statistics

According to M-P law, we can construct a test statistic by utilizing the eigenvalues of matrix $\sum$, without making restrictive assumptions about the distribution of elements in matrix ${{\varvec{M}}}$ to obtain the exact distribution of this statistic^26^. Linear Eigenvalue Statistics (LES) $\tau$, defined by the continuous test function $\varphi$:^27,28^, is given by

8 $$\begin{aligned} \tau (\varphi ,\Sigma ) = \sum \limits _{i = 1}^N {\varphi ({\lambda _i}) = {{\textrm{Tr}}}\varphi {{(}}} \Sigma ), \end{aligned}$$

where ${\lambda _i}$ is the *i*-th eigenvalue of $\sum$. Since each of ${\lambda _i}(\sum ),i = 1,2,\ldots ,N$ is a complex function of matrix $\sum$, $\tau$ is also a random variable concerning the sum of the function $\varphi$ of $\lambda$. Various test functions are proposed in^23^ as follows.

1. $${T_2}:\varphi (\lambda ) = 2{x^2} - 1$$
2. $${T_3}:\varphi (x) = 4{x^3} - 3x$$
3. $${T_4}:\varphi (x) = 8{x^4} - 8{x^2} + 1$$
4. $${T_{DET}}:\varphi (x) = \ln (x)$$
5. $${T_{LRT}}:\varphi (x) = x - \ln (x) - 1$$

In case that the elements of ${{\varvec{M}}}$ are independently identical distributed, the probability density function distribution of $\lambda$ is quite stable. So i.i.d. noise will not cause significant changes in the constructed LES. Therefore, using the random matrix-based LES to analyze time series data can effectively differentiate signals from Gaussian noise, enabling the detection of cyber attacks.

## Detection, differentiation and localization of hybrid attack

**Figure 2:**
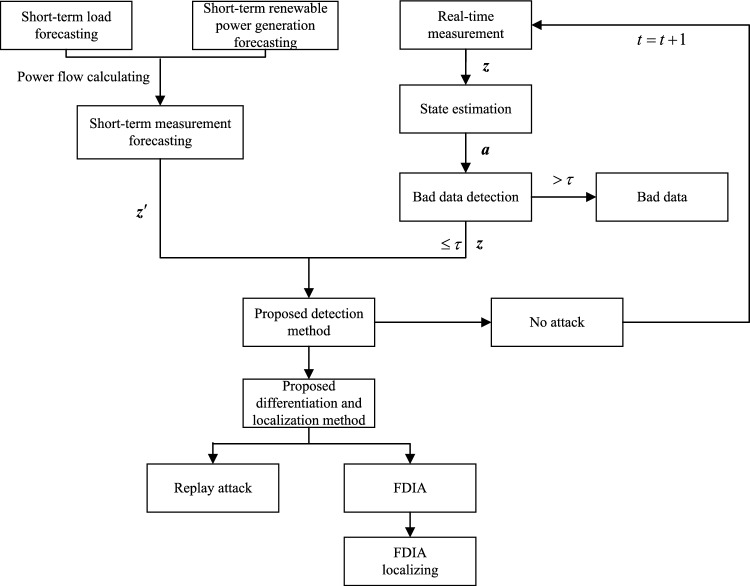
Proposed methodology for defending replay attack and FDIA.

We propose a comprehensive methodology shown in Fig. 2 to detect, differentiate and localize replay attack and FDIA.

(1) Forecast. By conducting load forecasting, renewable power generation forecasting and power flow calculation, predicted measurements ${{{\varvec{z}}}^{\prime}}$ are obtained.

(2) Detection. If no bad data are detected by BDD, real-time measurements ${{\varvec{z}}}$ will be sent to detection module based on measurements ${{{\varvec{z}}}^{\prime}}$ to detect hybrid attack.

(3) Differentiation and localization. Once a hybrid attack is detected, ${{\varvec{z}}}$ will be sent to the differentiation and localization module, aiming to identify the type of hybrid attack and further localize the attack if confirmed as FDIA.

In the subsequent sub-sections, we elaborate the models and algorithms for forecasting, detection, differentiation and localization, respectively.

### Short-term load forecasting and renewable power generation forecasting

It is critically important to improve the accuracy of the load forecast and renewable generation forecast, to achieve the effectiveness of the proposed detection method. In this paper, we apply the widely-used machine learning models to conduct day-ahead load forecast and renewable generation forecast, which are regarded as the ground truth for detection of hybrid attacks.

For day-ahead load forecast, we apply Random Forest (RF) and Long-short Term Memory (LSTM) neural network to construct an ensemble learning model with better performance. These methods have been widely used in Forecasting^29,30^. Specifically, we generate a training set by selecting similar days based on the weather and the trend of load changes. Then predicted power loads are obtained by these two methods respectively. Finally, weights based on the errors during forecast of the two models are settled to combine the prediction results.

As for renewable power generation forecast, we focus on wind power generation forecast as an example, for a mild research scope. Since the output of wind power is greatly influenced by speed and direction of wind, these two variables are selected as features to conduct the regression model. In this paper, a genetic algorithm optimized back propagation (GEN-BP) neural network is constructed for wind power generation forecast. The BP neural network has good performance in regression prediction, while optimizing the weight and bias using genetic algorithm can further improve forecasting performance^31^.

### Time-series analysis and detection

In this subsection, taking the predicted measurements as ground truth, we introduce the detection method based on RMT.

Suppose that the topology of the power system is fixed in short time period, after achieving power loads and renewable power generation forecasting results, the predicted measurements of power system can be obtained through power flow calculation. Then, we comprehensively securitize the deviation between actual measurements and predicted measurements and the chronological correlation of the measurements.

Let the deviation at *t* is ${{{\varvec{m}}}_t} = {{{\varvec{z}}}_t} - {{{\varvec{z'}}}_t}$. ${{\varvec{M}}} = {[{{{\varvec{m}}}_{t - T + 1}},{{{\varvec{m}}}_{t - T + 2}},\ldots ,{{{\varvec{m}}}_t}]^T}$ is a constructed random matrix with a sliding time window *T*. After standardizing each row of the constructed random matrix, the covariance matrix $\sum$ of ${{\varvec{M}}}$ is calculated as follows.

9 $$\begin{aligned} \sum = \frac{1}{N}{{\varvec{M}}}{{{\varvec{M}}}^T} \end{aligned}$$

By calculating the sum of a test function of the eigenvalues $[{\lambda _1},{\lambda _2},\ldots ,{\lambda _N}]$ of $\sum$, we can obtain the test statistic, namely, the LES $\tau$.

**Figure 3:**
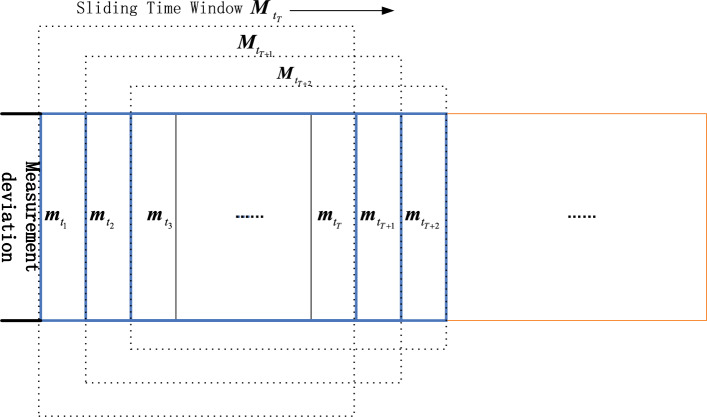
The designed sliding time window.

In order to analyze the time-series data, we need to obtain LES with dynamic changes over time. It is necessary to construct a series of random matrices at every time interval. To achieve the goal, the method of constructing a sliding time window is implemented. As shown in Fig. 3, a fix size of time window is sliding over time and the last column of the matrix in the time window is the deviation of the current moment.

If there is no attack, the differences between predicted measurements and real-time measurements containing gaussian noise are relatively small and LES changes insignificantly over time. If an attack occurs, the original distribution of the deviations in a time window changes and the temporal correlation of matrices at adjacent moments is broken, LES will appear significant fluctuations. We propose a statistic indicator denoted by

10 $$\begin{aligned} {\Delta \tau _t = {\tau _t} - {\tau _{t - 1}},} \end{aligned}$$

where $\tau _t$ and $\tau _{t - 1}$ represent the LES at the moment *t* and $t - 1$ respectively.

### Differentiation and localization

In this subsection, we investigate the differentiation and localization method based on multi-label CNN and the proposed detection method in “Section Time-series analysis and detection”.

#### Construction of location indicator

To improve the accuracy of FDIA localization, we construct a location indicator based on SVD decomposition and eigenvalue analysis. In traditional localization methods based on multi-label classification, measurements are directly used as input for training and predicting. The challenges that limit the accuracy of localization are twofold. (1) The significant differences in a meter at different moments reduce the effectiveness of training. (2) The significant differences between different meters under one FDIA reduce the accuracy for identifying the compromised meters under low-amplitude attacks. In response to challenge (1), by subtracting the real-time measurements from the predicted measurements, we set all normal measurements at the same level so as to highlight the compromised meter. Further in response to challenge (2), we propose a method to construct a location indicator based on the detection method in “Section Time-series analysis and detection” and matrix SVD decomposition to amplify the impact of FDIA on meters and utilize the indicator as input for CNN.

For a covariance matrix $\sum$ , we can decompose it as follows.

11 $$\begin{aligned} \sum = {{\varvec{v}}}\left[ {\begin{array}{*{20}{c}} {{\lambda _1}}&{}{}&{}{}\\ {}&{} \ddots &{}{}\\ {}&{}{}&{}{{\lambda _N}} \end{array}} \right] {{\varvec{u}}} \end{aligned}$$

12 $$\begin{aligned} \sum {{{\varvec{v}}}_k} = {\lambda _k}{{{\varvec{v}}}_k} \end{aligned}$$

where ${{\varvec{v}}}$ and ${{\varvec{u}}}$ represent the left and right eigenvector matrix respectively, ${\lambda _k}$ denotes the *k*th eigenvalue while ${v_k}$ is the corresponding eigenvector.

The contribution of the *i*-th row of ${{\varvec{v}}}$ to the eigenvalue ${\lambda _k}$ can be given by

13 $$\begin{aligned} {\sum \limits _{j = 1}^T {(\frac{{d{\lambda _k}}}{{d{\sum _{ij}}}})} ^2} = {v_{ik}}^2. \end{aligned}$$

The detailed derivation from Eq. (12) to (13) can be seen in^24^. we utilize the singular vector corresponding to the maximum singular value of constructed covariance matrix $\sum$ to construct the location indicator. The location indicator ${{\varvec{l}}}$ is given by

14 $$\begin{aligned} {l_i} = {\lambda _1}{v_{i1}}^2 \end{aligned}$$

where ${\lambda _1}$ is the maximum eigenvalue. Then ${{\varvec{l}}}$ of every measurement is set as the input for training and predicting of CNN.

#### CNN and label setting

**Figure 4:**
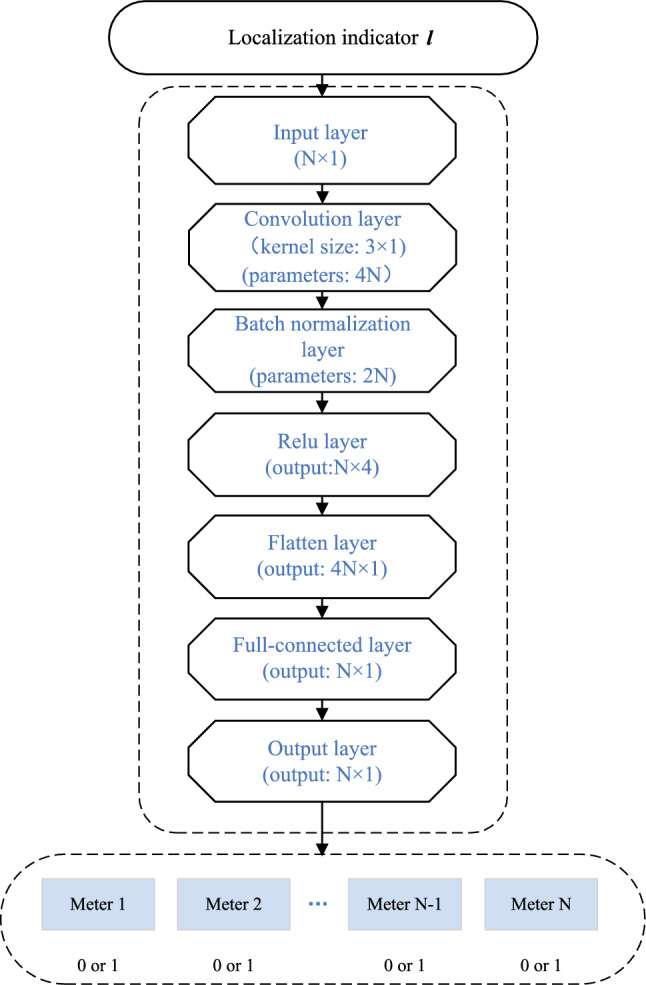
Structure of SVD-CNN.

In this subsection, we introduce the constructed multi-label CNN and label setting for differentiation and localization. The network structure of our constructed CNN is shown in Fig. 4. We denote the input (i.e., the location indicator), the labels (i.e., the meter compromised or not), the output (classification prediction of the CNN) as ${{\varvec{l}}} = ({l_1},{l_2},\ldots {l_n})$, ${{\varvec{y}}} = ({y_1},{y_2}\ldots {y_n})$ and ${{\varvec{\hat y}}} = ({\hat y_1},{\hat y_2}\ldots {\hat y_n})$, respectively. The label of meter *i* is determined according to the following rule

15 $$\begin{aligned} {y_i} = \left\{ \begin{array}{l} 1,{\mathrm{the\, meter\, }}i{\mathrm{ \,is\, compromised\, under\, FDIA}}\\ 0,{\mathrm{the\, meter\, }}i{\mathrm{\,is\, not\, compromised\, under\, FDIA}} \end{array} \right. \end{aligned}$$

Considering that replay attacks can be seen as a special normal state, we denote the label as ${{\varvec{y}}} = ({y_1},{y_2},\ldots {y_n}) = (0,0,\ldots 0)$ under replay attack. The output of CNN is continuous numbers around 0 and 1. We take the label closest to the output number as the output label.

The pseudo code of detection is shown in Algorithm 1. It is recommended that the threshold $\eta$ is greater than 95% of $\Delta \tau$ based on the normal operation of the power system, by evaluating historical operating data. The pseudo code of differentiation and localization is shown in Algorithm 2.

**Algorithm 1:**
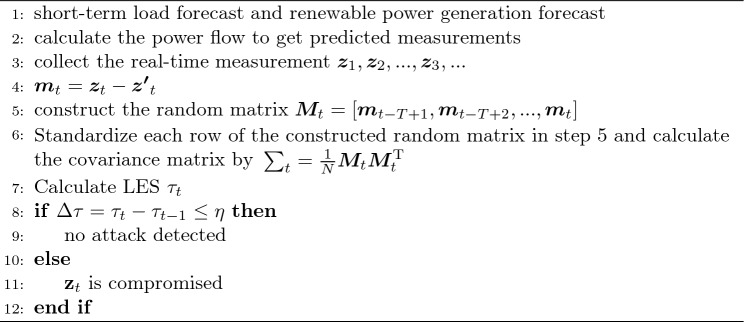
Detection.

**Algorithm 2:**
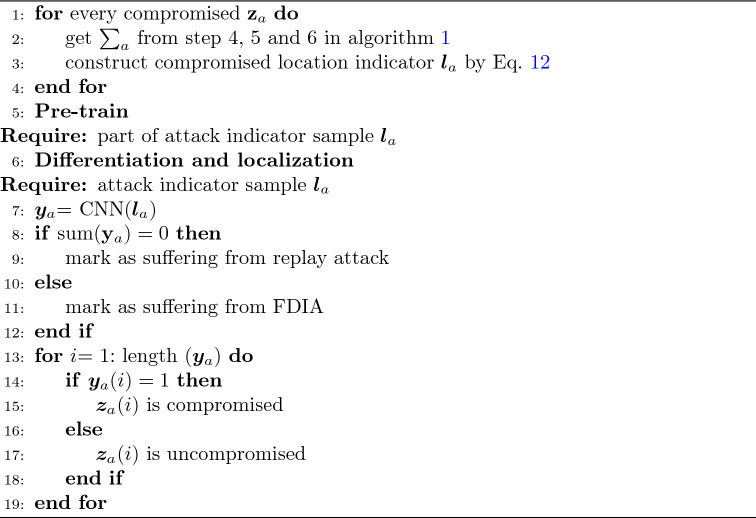
Differentiation and localization.

## Case studies

In this section, we evaluate the proposed defense methodology against replay attack and FDIA.

### Experimental setup

Our experiments are conducted by MATLAB 2021b on a personal computer with 16GB RAM, Intel i5-10210U CPU, and a NVIDIA GeForce MX110 GPU. The sections of forecast and localization are based on the machine learning and deep learning toolbox, while the section of power flow calculation utilize the MATPOWER toolbox. We first test and evaluate the effect of the detection method for replay attack and FDIA based on RMT through simulation experiments on the IEEE 14-bus system and IEEE 57-bus system. Then we verify the effectiveness of the proposed method of distinguishing replay attack from FDIA and the localization of FDIA.

### Short-term load and renewable generation forecast

In this section, we test the forecasting method of the proposed prediction model.

#### General setup of dataset

(1) Load forecast. The load data comes from some regions in a certain province in southern China which can be found online at https://github.com/c1emon/BP_Neural_Networks_for_power_load_forecasting. We select historical load information from 10 similar days within a month as the training set to forecast the last day of the month. The time interval is 15 min, so there are 960 sets of data in each model’s training set. The 96 sets of data on the day of prediction are set as the test set to verify the predicting results.

(2) Renewable power generation forecast. Our data comes from a wind farm which can be seen in^32^ . Taking a time interval of 10 min, a total of 4320 sets of data from the first 30 days of the month are set as the training dataset. A total of 144 sets of data from the 31st day are set as test dataset for regression prediction.

#### Result of short-term load forecast

**Figure 5:**
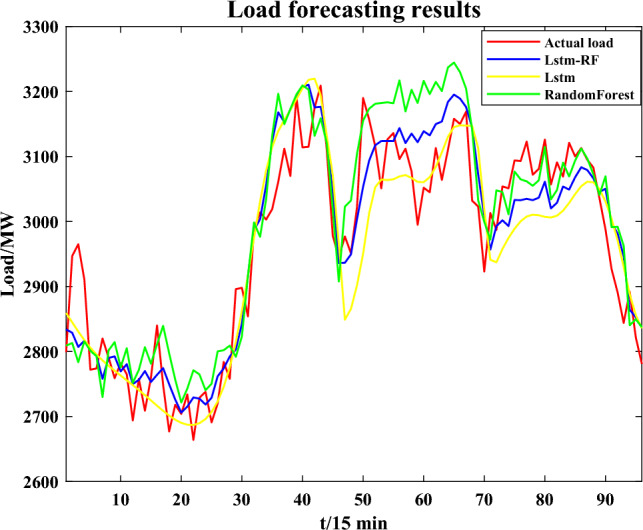
Load forecasting results.

We compare the effectiveness of proposed combination method for load forecast with using only a single model for forecasting the load of the 6th column on the last day of January. The result of load forecasting is shown in Fig. 5.

The indexes mean absolute percentage error (MAPE) and root mean square error (RMSE) are used to evaluate the prediction results. The error results based on different models are shown in Table 1. It is obvious to conclude that the proposed combined forecast model based on LSTM-RF has lower forecasting error.

**Table 1:** Error results of load forecast.

	RMSE (MW)	MAPE
RF	74.48	0.02016
LSTM	69.23	0.01874
LSTM-RF	60.41	0.01635

#### Result of short-term renewable power generation forecast

**Figure 6:**
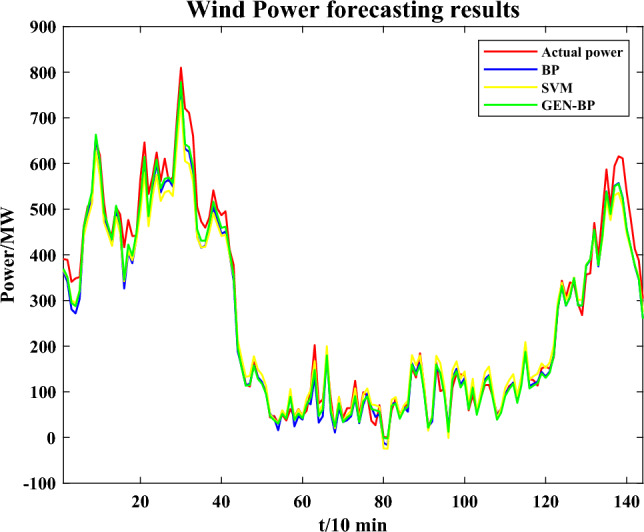
Wind power forecasting results.

The result of wind power forecasting is shown in Fig. 6.

Considering that wind power generation output may occur around 0, using MAPE as an index to evaluate errors is no longer appropriate. Mean absolute error (MAE) and RMSE are utilized to evaluate the error instead. The error results based on different models for wind power forecast are shown in Table 2.

**Table 2:** Error results of wind power forecast.

	RMSE (MW)	MAE (MW)
BP	32.60	0.02016
SVM	37.39	0.01874
GEN-BP	27.77	0.01635

It is obvious that the GEN-BP model achieves better prediction accuracy due to the superior performance of the BP network in regression prediction and the optimization of initial weights and biases by GEN.

### Case study 2: detection of hybrid attack

In this part, we study the performance of the proposed detection method in “Section Time-series analysis and detection” based on two aspects: the ability to detect hybrid attacks; the robustness to gaussian noise.

#### General setup of dataset

On the IEEE 14-bus system, there are a total of 14 buses and 20 transmission lines, i.e., a total of 34 measurements. On the IEEE 57-bus system, there are 57 buses and 80 transmission lines, i.e., a total of 137 measurements. The measurements are the active power injection of buses and branch flows. After obtaining the predicted loads and predicted wind power generations within a day, in order to match the sampling time of the two results and simulate a higher signal sampling frequency to meet the needs of a larger sliding time window, we fill in the predicted loads and predicted wind power generations according to the principle of equal distance to achieve a set of sampling data per minute. Due to the time interval of 15 min for load forecasting, from 0:00 to 23:45 in a day, 1425 sets of data were obtained. To balance the size of the data for test, the first 1425 sets of wind power generations are taken. On the IEEE 14-bus system, we normalize the data from columns 2 to 11 on the last day of January and the data from columns 2 to 5 on the last day of February, and then multiply them sequentially by the active load demand of the bus foundation. On the IEEE 57-bus system, the data from columns 2 to 11 on the last day of January and from columns 2 to 8 on the last day of July are included. The wind power generation data are normalized and then scaled by the active power generation of the second bus of IEEE 14-bus sysmtem, and the first bus of IEEE 57-bus system, respectively. Afterward, we perform power flow calculations to obtain predicted measurements. Due to the fact that measurement errors are generally assumed to be Gaussian distributions, and to avoid the sparsity of the constructed matrix, we add Gaussian white noise with a mean of 0 and deviation of 1% to the measurements. We set the size of a time window twice the number of meters to construct a random matrix, i.e., $T = 68$ on the IEEE 14-bus system and $T = 274$ on the IEEE 57-bus system. The test function 5 in “Section Linear eigenvalue statistics” is selected to construct LES. The construction of the attack is as follows:

1) replay attack: measurements from $t = 1$ min to $t = 680$ min are randomly selected as attack vectors to falsify real-time measurements from $t = 700$ min to $t = 1300$ min on the IEEE 14-bus system; measurements from $t = 1$ min to $t = 580$ min are randomly selected as attack vectors to falsify real-time measurements from $t = 600$ min to $t = 1100$ min on the IEEE 57-bus system.

2) FDIA: the attack vectors are constructed by the Eq. 2. The number of targeted state variables follows a discrete uniform [2, 4] distribution on the IEEE 14-bus system and a discrete uniform [9, 11] on the IEEE 57-bus system with a state variation of a uniform [10%, 20%] distribution for each bus.

#### Detection for replay attack

To study the effectiveness of our proposed method for replay attack detection, we inject continuous historical measurements from $t = 200$ min to $t = 300$ min into measurements from $t = 1100$ min to $t = 1200$ min on the IEEE 14-bus system and historical measurements from $t = 200$ min to $t = 300$ min into measurements $t = 1000$ min to $t = 1100$ min on the IEEE 57-bus system.

**Figure 7:**
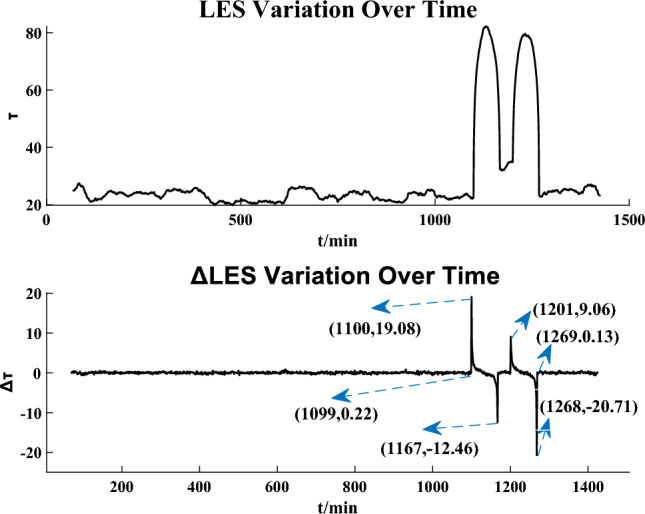
Replay attack detection result on the IEEE 14-bus system.

**Figure 8:**
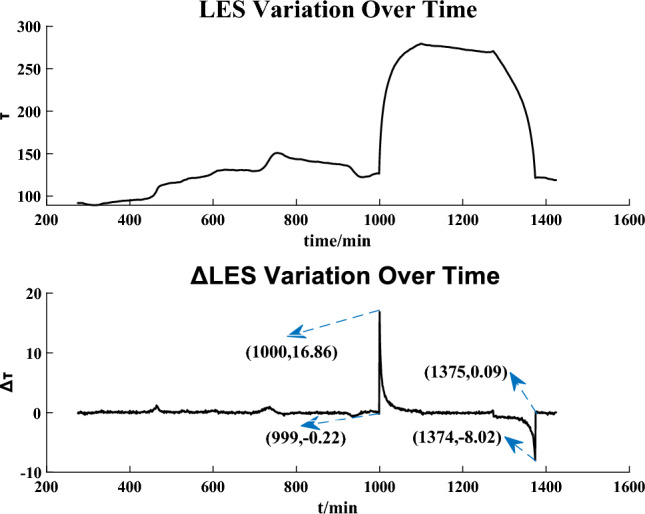
Replay attack detection result on the IEEE 57-bus system.

Figures 7 and 8 show the detection results on the IEEE 14-bus system and the IEEE 57-bus system, respectively. It is obvious that the curves of $\Delta \tau$ increase significantly at $t = 1100$ min and $t = 1000$ min (i.e., the moments that the attacks start) in the two figures, respectively. At $t = 1269$ min and $t = 1375$ min in Figs. 7 and 8 respectively, the sliding time windows completely slide out of the attacked time areas and $\Delta \tau$ regress to a lower value and keep in a low level after a trough.

It is obvious that the constructed LES is very sensitive to replay attacks and we can detect replay attacks by the sudden increase of $\Delta \tau$. It is noted that due to the different size relationship between the sliding time window and attack duration, the trends of the curves in Figs. 7 and 8 are not the same, but this will not affect the real-time detection.

Further to demonstrate the performance of our proposed detection method for filtering measurement noise, We set different detection methods for comparison. The comparable detection methods are set as follows

1. our proposed method based on prediction and LES (PD-LES)
2. utilizing $\Delta {\textrm{RMSE}}$ of the differences from predicted measurement and real-time measurement as the test statistic (RMSE-PD)
3. utilizing the actual measurements to construct a random matrix and obtain $\Delta {\textrm{LES}}$ as the test statistic (AM-LES)

**Figure 9:**
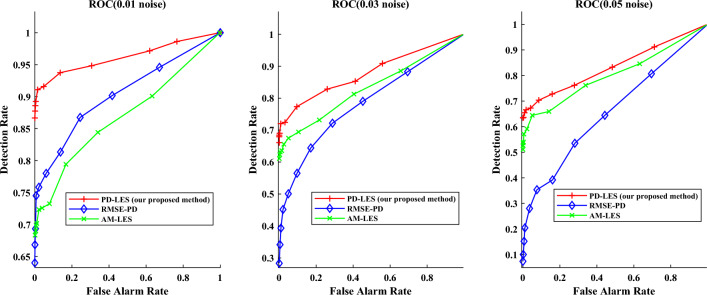
ROC of different detection method on the IEEE 14-bus system.

**Figure 10:**
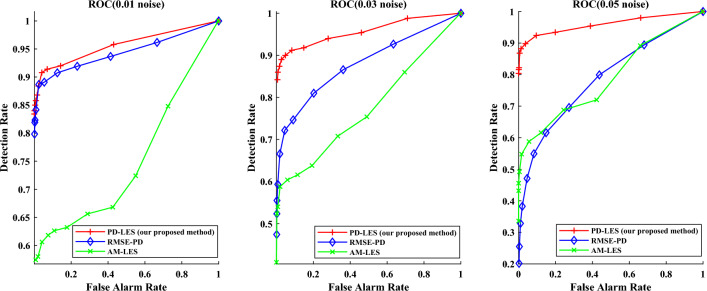
ROC of different detection method on the IEEE 57-bus system.

We use receiver operating characteristic curve (ROC) to show the effectiveness. To ensure real-time detection, we focus on the detection effectiveness of the attack-launching moment. As the computational efficiency of detection shown in Table 3, the time for calculating $\Delta \tau$ is very short, which can meet the requirement of online detection. We repeat attacks for 1200 times on the IEEE 14-bus system while 500 times on the IEEE 57-bus system. Additionally, we add additional magnitudes of noise (Gaussian distribution with 0 mean and variance of 0.03, and 0.05 respectively) to imitate measurement errors. The ROCs of detection on the IEEE 14-bus system and IEEE 57-bus system are shown in Figs. 9 and 10 respectively. The detection rates at 0 false alarm rate for these 3 methods as the noise increases on the IEEE 14-bus system and IEEE 57-bus system are shown in Tables 4 and 5 respectively.

**Table 3:** Computational efficiency of detection.

Time(s) for pre-prediction		Time(s) for calculating $\Delta \tau$	
Load	Wind power generation	IEEE 14-bus system	IEEE 57-bus system
35.2	45.5	0.002	0.009

**Table 4:** Detection rate on the IEEE 14-bus system.

Noise amplitude	PD-LES (%)	RMSE-PD (%)	AM-LES (%)
0.01	86	64	68
0.03	65	29	60
0.05	62	9	50

**Table 5:** Detection rate on the IEEE 57-bus system.

Noise amplitude	PD-LES (%)	RMSE-PD (%)	AM-LES (%)
0.01	84	80	56
0.03	83	48	40
0.05	80	20	32

From the Figs. 9 and 10, it is obvious that the detection rate of PD-LES is much higher than the other two methods, which means it is easy to choose a threshold to balance the false alarm rate and detection rate, so as to achieve a higher detection rate at low false alarm rate. More importantly, it is obvious that as the noise amplitude increases, the decrease of detection rate of RMSE-PD is larger than that of PD-LES and AM-LES. In other words, our proposed detection method based on RMT is robust by filtering measurement noise.

It can be seen that the LES of random matrix is very sensitive to the noise that converges to i.i.d., so it is equivalent to play a good role in filtering measurement noise. This can also explain the phenomenon that the decrease in detection rate is more affected by the increase of noise amplitude for RMSE-PD. Both PD-LES and AM-LES utilize RMT, while RMSE-PD does not. In addition, the detection rate of PD-LES is much better than AM-LES. This is because, as the power system operates dynamically, the distribution of real-time measurements within a sliding time window in AM-LES is more irregular, while the difference between predicted measurements and real-time measurements can be approximated as white noise, the statistical pattern within a sliding window is more sensitive to a cyber attack, leading to enhanced detection accuracy. Hence, measurement forecasting is an essential aspect of our proposed approach. Furthermore, with the increase of the size of a time window, the distribution of data is more complex in a time window, which makes the detection effect of AM-LES on the IEEE 57-bus system worse than that on the IEEE 14-bus system.

#### Detection for FDIA

**Figure 11:**
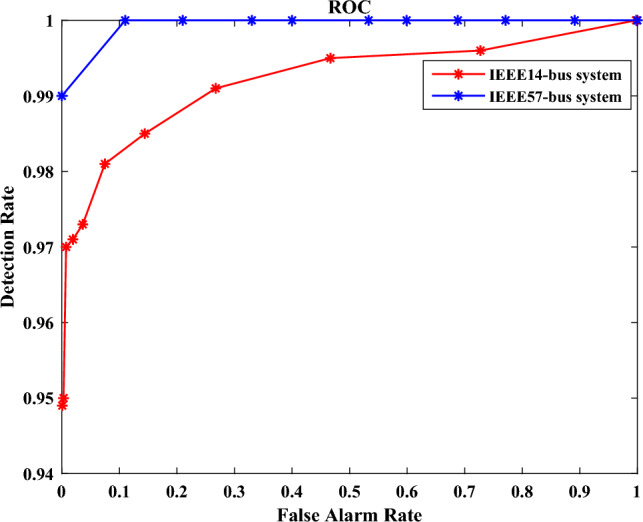
ROC of proposed method for FDIA detection.

For FDIA, the detection rate is also quite high. Figure 11 shows the ROC of proposed method for FDIA detection under 0.05 noise. From the figure, it can be seen that even at high noise level, when the false alarm rate is equal to 0, the FDIA detection rate of our proposed has reached over 94%. It is concluded that our proposed method can achieve high detection rate for FDIA, even given few number of targeted states and small changes of state variables.

### Case study 3: differentiation and localization

In this section, we study the performance of our proposed method in “Section Differentiation and localization” for differentiation and localization to validate the high sensitiveness to compromised meters of our constructed location indicator.

#### General setup of dataset

The construction of attack vectors is the same as that in “Section General setup of dataset” of “Section Case study 2: detection of hybrid attack”. For CNN, we use the gradient descent algorithm to train the network, with 30 training samples each time. The total number of training times is 900. The learning rate is 0.01 and it becomes 0.005 after 400 times’ training. We have constructed 3000 conventional FDIA samples and 3000 replay attack samples. After shuffling the sample order, select 4500 samples as the training set and 1500 samples as the testing set.

#### Location indicator

**Figure 12:**
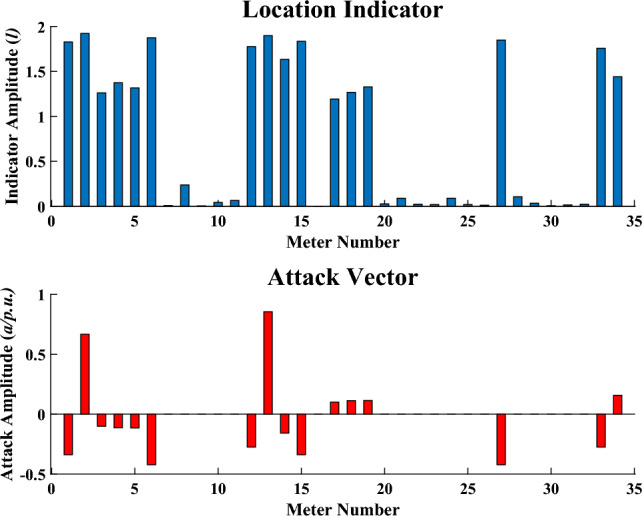
Location indicator for FDIA.

**Figure 13:**
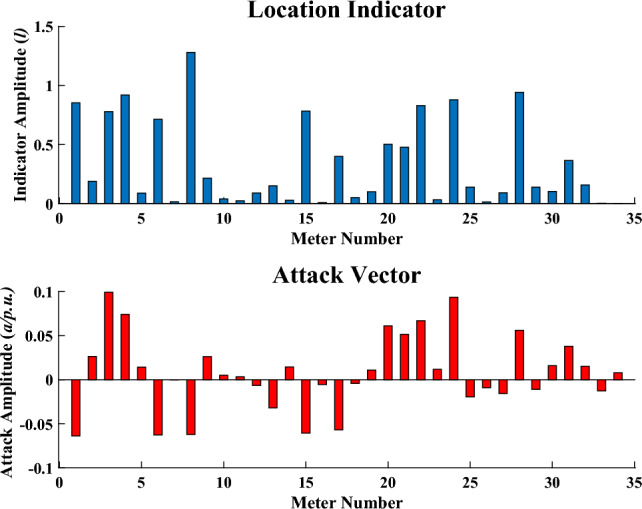
Location indicator for replay attack.

The result of our proposed location indicator for FDIA is shown in Fig. 12 while the location indicator of the proposed method for replay attack is shown in Fig. 13.

In Fig. 12, it can be seen that for the meters which are not attacked, the values of the indicators are very small. However, for the meters under attack, even if the meter with the lowest attack amplitude, the corresponding location indicator is far greater than that of the uncompromised meters. By this way, the differences between compromised meters and uncompromised meters are amplified. For replay attacks in Fig. 13, all measurements are attacked, which lead to the relatively centralized distribution of location indicators. Therefore, the classifier can easily differentiate between the two attack types by analyzing the variation in location indicator distribution.

#### Result of differentiation and localization

In our proposed model, the computational efficiency of differentiation and localization is shown in Table 6. In order to further validate the improvement of the proposed data processing method on localization performance, we set up a comparative experiment of CNN-based classification. Besides the traditional method using real-time measurements directly as input to CNN, we also use the difference between actual measurements and predicted measurements as input (DIF-CNN) for comparison. What’s more, we compare CNN with traditional machine learning methods decision trees with location indicator as input (SVD-DT) to demonstrate its superiority in classification performance.

In order to utilize decision tree (a conventional method to solve the single-label multi-class problem) to solve the multi-label binary classification problem, inspired by^14^, the multi-label dataset with *N* binary labels is converted into a single-label dataset with ${2^N}$ categories. Due to the fact that some measurements are related and the number of targeted state variables is limited, there are still hundreds of categories on the IEEE 14-bus system after deleting the categories that have never appeared. On the IEEE 57-bus system, the number of categories is much more.

In this paper, we employ accuracy, precision, recall, and F1 of the predicted results to evaluate the performance of the classifiers. It is noted that, the definitions of true positive (TP), false positive (FP), true negative (TN) and false negative (FN) for differentiation and localization are defined in Tables 7 and 8, respectively. The evaluation indicators are defined in Eqs. (16), (17), (18) and (19) respectively. The differentiation and localization results on the IEEE 14-bus system are shown in Tables 9 and 10 respectively.

**Table 6:** Computational efficiency of differentiation and localization.

	Time(s) for pre-training	Time(s) for differentiation and and localization
IEEE 14-bus system	12.3	0.015
IEEE 57-bus system	15.2	0.021

**Table 7:** Definitions of TP, FP, TN and FN for differentiation.

	FDIA (predicted)	Replay attack (predicted)
FDIA (real)	TP	FN
Replay attack (real)	FP	TN

**Table 8:** Definitions of TP, FP, TN and FN for localization.

	Compromised meter(predicted)	Uncompromised meter(predicted)
Compromised meter(real)	TP	FN
Uncompromised meter(real)	FP	TN

16 $$\begin{aligned} {\textrm{Accuracy}}&= \frac{{TP + TN}}{{TP + TN + FP + FN}} \end{aligned}$$

17 $$\begin{aligned} {\textrm{Precise}}&= \frac{{TP}}{{TP + FP}} \end{aligned}$$

18 $$\begin{aligned} {\textrm{Recall}}&= \frac{{TP}}{{TP + FN}} \end{aligned}$$

19 $$\begin{aligned} {\textrm{F1}}&= \frac{{2 \times {\textrm{Precise}} \times {\textrm{Recall}}}}{{{\textrm{Precise}} + {\textrm{Recall}}}} \end{aligned}$$

As can be seen in Table 9, in term of the differentiation, by using the difference between the actual measurements and the predicted measurements as input for CNN, the four indexes enhance significantly to near 100%. Further using the location indicator readings as input, the improvement is little, and the gap between SVD-CNN and SVD-DT is not very large. The reason is that differentiating the type of attacks is a binary classification problem, and the distribution of the measurement readings of the two types of attack is significantly different, so the traditional machine learning model, decision tree, can also achieve results that are quite accurate.

**Table 9:** Results of differentiation on the IEEE 14-bus system.

Method	Accuracy (%)	Precise (%)	Recall (%)	F1 (%)
CNN	85.13	77.03	98.90	86.61
DIF-CNN	99.60	99.20	100	99.60
SVD-CNN	100	100	100	100
SVD-DT	98.47	97.29	99.74	98.50

It can be seen in Table 10 that, compared with SVD-DT, SVD-CNN shows better performance. The reason is that, besides the superiority of SVD-CNN in classification, the method of multi-label classification takes the correlation of each label into account. So multi-label CNN performs well in localization. Specifically, by subtracting the real-time measurements from the predicted measurements, we set all normal measurements at the same level so as to highlight the compromised meter, so the performance of DIF-CNN improves a lot compared to CNN. What’s more, SVD-CNN has the most significant improvement in the recall, which means that more compromised measurements are identified. This also proves the effectiveness of our proposed method in amplifying the difference between compromised meters and uncompromised meters.

**Table 10:** Results of localization on the IEEE 14-bus system.

Method	Accuracy (%)	Precise (%)	Recall (%)	F1 (%)
CNN	85.69	99.24	73.31	84.33
DIF-CNN	96.85	99.94	93.99	96.88
SVD-CNN	99.26	99.90	98.72	99.31
SVD-DT	78.62	79.83	84.72	82.20

Tables 11 and 12 show the result of differentiation and localization on the IEEE 57-bus system respectively. For differentiation, SVD-CNN can also achieve good performance and the four indexes are all near 100%. For localization, because there are numerous categories which limit the effectiveness of training, the performance of SVD-DT is poor. What’s more, Our proposed method has better performance in localization especially a higher index of recall.

**Table 11:** Results of differentiation on the IEEE 57-bus system.

Method	Accuracy (%)	Precise (%)	Recall (%)	F1 (%)
CNN	89.47	82.40	99.86	90.29
DIF-CNN	99.93	100	99.87	99.93
SVD-CNN	99.67	100	99.33	99.67
SVD-DT	99.73	100	99.46	99.73

**Table 12:** Results of localization on the IEEE 57-bus system.

Method	Accuracy (%)	Precise (%)	Recall (%)	F1 (%)
CNN	88.82	95.84	75.19	84.27
DIF-CNN	93.77	98.19	86.02	91.70
SVD-CNN	95.98	98.20	91.67	94.82
SVD-DT	53.77	39.87	36.84	38.20

## Conclusion

Replay attack and FDIA both disrupt the normal operation of CPPS by compromising the SCADA system of the power system. Replay attack utilizes historical measurements to falsify real-time measurements while FDIA injects biases into the measurements.

In order to defend against the replay attack and FDIA, following work has been done. (1) We use the RMT to construct LES to filter the noise in the time series predicted measurements to achieve the goal of detecting replay attack. (2) We propose an SVD-CNN method to differentiate between FDIA and replay attacks and localize FDIA. Simulations have proven that our methods for detection show robustness to measurement noise. What’s more, the accuracy of the our proposed method in differentiation is close to 100%; compared to CNN, the recall rate of the proposed method for FDIA localization has increased from 73.31 to 98.72% on the IEEE 14-bus system and from 75.19 to 91.67% on the IEEE 57-bus system, respectively. We achieve identification of compromised measurements with higher probability.

It should be noted that on larger systems, wider time windows are required, which means that the frequency of data sampling needs to be further increased. In the future, we will study the detection and differentiation of other different types of cyber attacks.

## Data Availability

The data analysed during the current study are included in this article.

## References

[1] Mo, Y. & Sinopoli, B. Secure control against replay attacks. In *2009 47th Annual Allerton Conference on Communication, Control, and Computing (Allerton)*, Monticello, IL, USA, 911–918. 10.1109/ALLERTON.2009.5394956 (2009).

[2] Mo Y, Chabukswar R, Sinopoli B (2014). Detecting integrity attacks on SCADA systems. IEEE Trans. Control Syst. Technol..

[3] Tran, T. -T., Shin, O. -S. & Lee, J.-H. Detection of replay attacks in smart grid systems. In *2013 International Conference on Computing, Management and Telecommunications (ComManTel)*, Ho Chi Minh City, Vietnam, 298–302. 10.1109/ComManTel.2013.6482409 (2013).

[4] Irita, T., & Namerikawa, T. Detection of replay attack on smart grid with code signal and bargaining game. In *2017 American Control Conference (ACC)*, Seattle, WA, USA, 2112–2117. 10.23919/ACC.2017.7963264 (2017).

[5] Zhao, J., Wang, J. & Yin, L. Detection and control against replay attacks in smart grid. In 12th *International Conference on Computational Intelligence and Security (CIS)*, Wuxi, China, 624–627. 10.1109/CIS.2016.0151 (2016).

[6] Liu, Y., Ning, P. & Reiter, M.K. False data injection attacks against state estimation in electric power grids. In *Proceedings of the 16th ACM Conference on Computer and Communications Security* 09, 21–32 (2009). 10.1145/1653662.1653666.

[7] Liu X, Bao Z, Lu D, Li Z (2015). Modeling of local false data injection attacks with reduced network information. IEEE Trans. Smart Grid.

[8] Lakshminarayana S, Kammoun A, Debbah M, Poor HV (2021). Data-driven false data injection attacks against power grids: A random matrix approach. IEEE Trans. Smart Grid.

[9] Siu JY, Kumar N, Panda SK (2022). Command authentication using multiagent system for attacks on the economic dispatch problem. IEEE Trans. Ind. Appl..

[10] Bi J, Luo F, Liang G, Yang X, He S, Dong ZY (2023). Impact assessment and defense for smart grids with FDIA against AMI. IEEE Trans. Netw. Sci. Eng..

[11] Jafari M, Ashiqur Rahman M, Paudyal S (2023). Optimal false data injection attacks against power system frequency stability. IEEE Trans. Smart Grid.

[12] Luo X, He J, Wang X, Zhang Y, Guan X (2023). Resilient defense of false data injection attacks in smart grids via virtual hidden networks. IEEE Internet Things J..

[13] Wang D, Wang X, Zhang Y, Jin L (2019). Detection of power grid disturbances and cyber-attacks based on machine learning. J. Inf. Secur. Appl..

[14] Wang S, Bi S, Zhang Y-JA (2020). Locational detection of the false data injection attack in a smart grid: A multilabel classification approach. IEEE Internet Things J..

[15] Qin Z, Lai Y (2022). Detection and localization of coordinated state-and-topology false data injection attack by multi-modal learning. J. Electr. Eng. Technol..

[16] Chaojun G, Jirutitijaroen P, Motani M (2015). Detecting false data injection attacks in AC state estimation. IEEE Trans. Smart Grid.

[17] Singh SK, Khanna K, Bose R, Panigrahi BK, Joshi A (2018). Joint-transformation-based detection of false data injection attacks in smart grid. IEEE Trans. Ind. Informat..

[18] Zhang Z, Hu J, Lu J, Cao J, Alsaadi FE (2022). Preventing false data injection attacks in LFC system via the attack-detection evolutionary game model and KF algorithm. IEEE Trans. Netw. Sci. Eng..

[19] Wang Y, Zhang Z, Ma J, Jin Q (2022). KFRNN: An effective false data injection attack detection in smart grid based on Kalman filter and recurrent neural network. IEEE Internet Things J..

[20] Deng, Y., Zhu, K., Wang, R. & Wan, Y. Real-time detection of false data injection attacks based on load forecasting in smart grid. In *2019 IEEE International Conference on Communications, Control, and Computing Technologies for Smart Grids (SmartGridComm)*, Beijing, China, 1–6. 10.1109/SmartGridComm.2019.8909811 (2019).

[21] Du, J., Wang, C., Peng, C. & Hou, Y. A random matrix based high-resistance grounding fault detection method using PMU measurements. In *IEEE PES Innovative Smart Grid Technologies Conference Europe (ISGT-Europe)*, Ljubljana, Slovenia **2016**, 1–5. 10.1109/ISGTEurope.2016.7856252 (2016).

[22] He X, Qiu RC, Ai Q, Chu L, Xu X, Ling Z (2016). Designing for situation awareness of future power grids: An indicator system based on linear eigenvalue statistics of large random matrices. IEEE Access.

[23] He X, Ai Q, Qiu RC, Huang W, Piao L, Liu H (2017). A big data architecture design for smart grids based on random matrix theory. IEEE Trans. Smart Grid.

[24] Ling Z, Qiu RC, He X, Chu L (2021). A new approach of exploiting self-adjoint matrix polynomials of large random matrices for anomaly detection and fault location. IEEE Trans. Big Data.

[25] Chang C-B, Dunn K-P (2016). Applied State Estimation and Association.

[26] Qiu R, Wicks M (2013). Cognitive Networked Sensing and Big Data.

[27] Lytova A, Pastur L (2009). Central limit theorem for linear eigenvalue statistics of random matrices with independent entries. Ann. Probab..

[28] Shcherbina M (2011). Central Limit Theorem for linear eigenvalue statistics of the Wigner and sample covariance random matrices. J. Math. Phys. Anal. Geom..

[29] Wang P (2022). An online electricity market price forecasting method via random forest. IEEE Trans. Ind. Appl..

[30] Ciechulski T, Osowski S (2021). High precision LSTM model for short-time load forecasting in power systems. Energies.

[31] Xu, M., Ma, C. & Han, X. Influence of Different Optimization Algorithms on Prediction Accuracy of Photovoltaic Output Power Based on BP Neural Network. In *41st Chinese Control Conference (CCC)*, Hefei, China, **2022**, 7275–7278. 10.23919/CCC55666.2022.9902165 (2022).

[32] Goh, H. H., He, R., Zhang, D., Liu, H., Dai, W., Lim, C. S., Kurniawan, T. A., Teo, K. T. K. & Goh, K. C. A multimodal approach to chaotic renewable energy prediction using meteorological and historical information. Mendeley Data, V1. 10.17632/gpytf8x3ys.1 (2022).

